# The effect of resveratrol on the expression of MDR1 gene in leukemic lymphoblast’s of acute lymphoblastic leukemia patients

**Published:** 2015

**Authors:** Abbas Ali Hosein poor Feyzi, Majid Farshdousti Hagh, Tohid Ebadi, Karim Shams Asanjan, Aliakbar Movasagpoor Akbari, Mehdi Talebi, Behzad Emadi

**Affiliations:** 1 Hematology and Oncology Research Center, Tabriz University of Medical Sciences, Tabriz, Iran; 2 Department of Hematology, Faculty of Medicine, Tabriz University of Medical Sciences, Tabriz, Iran.; 3 Department of Microbiology, Faculty of Medicine, Iran University of Medical Science, Tehran, Iran

**Keywords:** Resveratrol, Multidrug resistance gene, Leukemic lymphoblast, ALL

## Abstract

**Background:**

Chemotherapy plays a very important role in the treatment of leukemia but the resistance properties of the lymphoblasts limit the effect of chemotherapy. One of the main mechanisms of resistance to chemotherapy is the increased expression of MDR1 gene. The aim of this study was to explore the effect of resveratrol on the expression of MDR1 gene in leukemic lymphoblast of new cases of acute lymphoblastic leukemia (ALL) patients in vitro.

**Methods:**

Separation of lymphoblasts of 5 new case ALL patients from peripheral blood was performed by ficoll density gradient centrifugation. Lymphoblasts were cultured in RPMI 1640 medium. Lymphoblasts were treated with 50µmol/L resveratrol for 48 h. Total RNA was extracted with guanidine isothiocyanate. RNA was converted to cDNA. Real time PCR was used to detect mRNA expression of MDR1.

**Results:**

The results of gene detection showed that the expression of MDR1 did not change significantly in the patients however, in one patient expression of MDR1 increased upon treatment with resveratrol.

**Conclusion:**

The results of this study did not support resveratrol as a compound to reverse multidrug resistance in leukemic lymphoblasts.

Acate lymphoblastic leukemia (ALL) is the most common malignancy in children (1). Chemotherapy has an important role in the treatment of malignancies, but resistance properties of lymphoblasts may decrease the effect of chemotherapy. Lymphoblasts can develop resistance to the drugs used in chemotherapy. Furthermore, some leukemia become resistant to other drugs that their functions are not related. This phenomenon is multidrug resistance (MDR) (2). P-glycoprotein which is an ATP binding glycoprotein is organized in two transmembrane domains and coded by the MDR1 gene (3, 4). The hydrolysis of ATP enables the conformational changes in P-glycoprotein leading to the extrusion of drugs out of the cell (4).Some natural and synthetic substances have been tested to overcome multidrug resistance(5). These include calcium channel blockers (verapamil) (6). However, these substances were not successful in clinical practice because of their severe side effects in vivo (7). Resveratrol acts as anti-inflammatory drug in the form of powdered root Polygonum cuspidatum (8). Resveratrol prevents plants from being aggressive by fungal and other forms. The skin of the grape contains higher level of resveratrol (9).

Resveratrol has a dose-dependent effect on mitotic and apoptotic activity of endothelial and tumor cell lines, while the low dose of resveratrol enhance cell proliferation; higher doses induce apoptosis and decrease mitotic activity (10). Resveratrol is known to have potent anti-inflammatory and antioxidant effects and inhibits the growth of a variety of cancer cells (11). In this research, we evaluated the effect of resveratrol on the expression of MDR1 gene in leukemic lymphoblast’s of new case ALL patients in vitro.

## Methods

5 new ALL patient cases were enrolled in the study after filling out the informed consent form. All patients had more than 100x10³/µl white cells in peripheral blood with up to 80% blasts. Samples were obtained before starting chemotherapy. Mononuclear cells were separated from 3ml of peripheral blood of the patients with the use of fycol and centrifuge.

**Cell culture: **Cells were grown in RPMI 1640 medium (Gibco, Invitrogen, USA), under sterile condition. Cells were incubated in a humidified 5% CO2 incubator at 37°C. The lymphoblasts of each patient were divided into two parts and cultured in separated cell culture T-25 flasks in which one of them was the group treatment with resveratrol and the control group.

**Treatment of the cells with resveratrol: **Resveratrol with the concentrations of 50 moll/L was added to the flasks then incubated in 37°C and 5% CO2 incubator. After 48 h of incubation, the total RNA was extracted then reversely transcribed into cDNA using an ExScriptRT reagent kit (Bioneer). primers: MDR1(87bp) sense 5´-CGGGAGCAGTCATCTGTGGT-3´ antisense 5´-CAAAGAGAGCGAAGCGGCTG-3´; β-actin (160 bp) sense 5´-AGA ACA TCA TCC ATG CAT CCA-3´, antisense 5´-GCC TGC TTC ACC ACC TTC TTG-3´. Real time-PCR conditions were set as follows: 94° C for 10 min, 45 cycles at 94 °C for 30 s, 58 °C for 30 s, and 72° C for 35 s; followed by extension at 72 °C for 10 min. All the data were processed with SPSS13.0. One-factor variance analysis (ANOVA) was used in the comparison of several concentrations and the significance of difference was indicated as p<0.05.

## Results

The relative expression of MDR1 gene was analyzed by 

**Figure 1:**
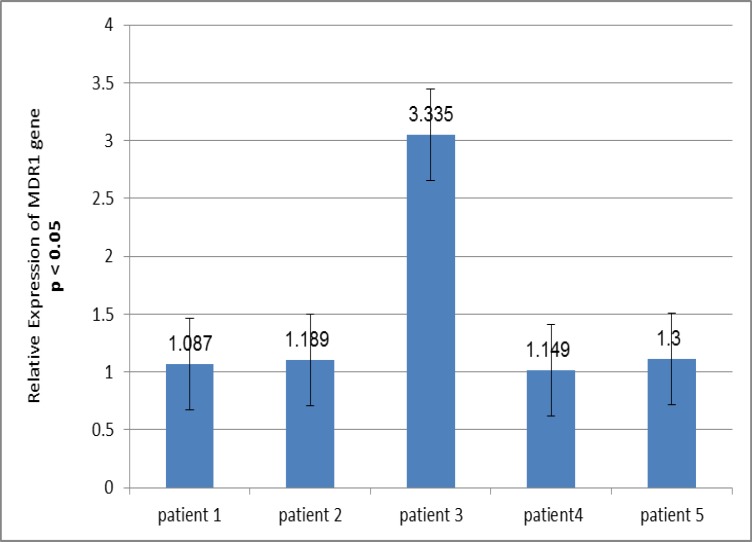
Effect of resveratrol on the expression of MDR1 gene in leukemic lymphoblasts

Real-time-PCR. After 48 h treatment with 50 µmol/L of resveratrol, the expression of MDR1 gene was not changed significantly in lymphoblasts in 4 patients and was in the range of 1.087 to 1.3 times, but in one patient increased upon treatment (3.335 times) (fig 1).

## Discussion

MDR1, express P-glycoprotein, a transmembrane carrier that exports hydrophobic substrates and most chemotherapy drugs out of the cells especially tumor cells. A growing body of literature indicates that resveratrol, a plant constituent enriched with the skin of grapes (12), is one of the promising agents for the prevention of heart disease. Similarly to other polyphenols resveratrol exerts an antioxidant effect (13). In addition resveratrol acts as an antioxidant (10, 14). Belaszenda demonstrated that resveratrol has a dose-dependent effect on mitotic and apoptotic activity of endothelial and tumor cell lines. They showed that low doses of resveratrol enhance cell proliferation; higher doses induce apoptosis and decrease mitotic activity. Fang Guan assessed the effect of resveratrol on the expression of MDR1 gene in human oral epidermis KBv200 cell line and showed that resveratrol could down-regulate MDR1 gene (15). But the results of our study showed that the expression of MDR1 gene was not changed significantly in the lymphoblasts of 4 patients and in one patient increased upon treatment. The reason for these unexpected results is not entirely clear. It may also be because the concentration used for treatment, was low. Also 48 h treatment with resveratrol may be not sufficient for resveratrol to take effect on gene expression. Furthermore, we supplemented the culture medium with fetal bovine serum which may interfere with gene expression. Furthermore, the MDR1 gene expression in one patient is very high in comparison to other patients as you can see in figure 1 (3.335 times). This high change may be the under effect of genetic differences or special translocations. Resveratrol may have different effects in the upper concentrations that need to be assessed in the future.

In conclusion, we did not find any sign in support of resveratrol as a compound reversing multidrug resistance in leukemic lymphoblasts.

## Notes

**Funding:** This research was funded by the Hematology and Oncology Research Center of Tabriz University of Medical Sciences.
